# Can Neglected Tropical Diseases Compromise Human Wellbeing in Sex-, Age-, and Trait-Specific Ways?

**DOI:** 10.1371/journal.pntd.0004489

**Published:** 2016-04-14

**Authors:** David C. Geary

**Affiliations:** Department of Psychological Sciences, Interdisciplinary Neuroscience, University of Missouri, Columbia, Missouri, United States of America; University of Iowa College of Public Health, UNITED STATES

## Abstract

Traits that facilitate competition for reproductive resources or that influence mate choice have evolved to signal resilience to infectious disease and other stressors. As a result, the dynamics of competition and choice can, in theory, be used to generate predictions about sex-, age-, and trait-specific vulnerabilities for any sexually reproducing species, including humans. These dynamics and associated vulnerabilities are reviewed for nonhuman species, focusing on traits that are compromised by exposure to parasites. Using the same approach, sex-, age-, and trait-specific vulnerabilities to parasitic disease are illustrated for children’s and adolescent’s physical growth and fitness. Suggestions are then provided for widening the assessment of human vulnerabilities to include age-appropriate measures of behavioral (e.g., children’s play) and cognitive (e.g., language fluency) traits. These are traits that are likely to be compromised by infection in age- and sex-specific ways. Inclusion of these types of measures in studies of neglected tropic diseases has the potential to provide a more nuanced understanding of how these diseases undermine human wellbeing and may provide a useful means to study the efficacy of associated treatments.

The World Health Organization [1] reports that the most common neglected tropical diseases (NTDs) compromise the wellbeing of about 1.4 billion people worldwide, including the poor, in many otherwise wealthy nations [2]. These diseases exact a substantial personal, social, and economic toll on their victims and the larger communities within which the afflicted reside [3]. These costs are well documented but may not fully capture the overall impact of NTDs, because even mild levels of disease burden may have sex-, age-, and trait-specific effects that have largely gone unrecognized. In the final section, I provide discussion of core physical, behavioral, and cognitive traits in boys and girls and in men and women that may show heighted sex-specific sensitivity to NTDs and suggestions on how to assess these traits. I begin with an introduction to the basic biological principles underlying the concept of sex-, age- and trait-specific vulnerabilities and briefly illustrate them in a few nonhuman species. The first section thus provides the theoretical foundation that enables the identification of vulnerable traits and describes why this foundation is applicable to all sexually reproducing species.

## Evolution of Vulnerable Traits

The potential for sex-, age-, and trait-specific consequences of parasitic infection have been well studied in nonhuman species and follow from the evolution and expression of condition-dependent traits [4–6], as elaborated below. These traits are common in sexually reproducing species and signal to conspecifics (members of their own species) the degree to which individuals have been exposed to one or more natural stressors—parasites, poor nutrition, and social stress—and their ability to cope with them. The evolution of these traits is driven by sexual and social selection [7,8]; specifically, traits that facilitate social competition for mates or other important resources (e.g., food, nesting sites) or are correlated with these traits and that influence mate choice. The traits themselves—for instance, antler size or bird song—can differ across the sexes and even across closely related species, but should be identifiable a priori for each sex, species, and developmental period (e.g., prenatal, adulthood) with a sufficient understanding of the dynamics of competition and choice in the species.

**Fig 1:**
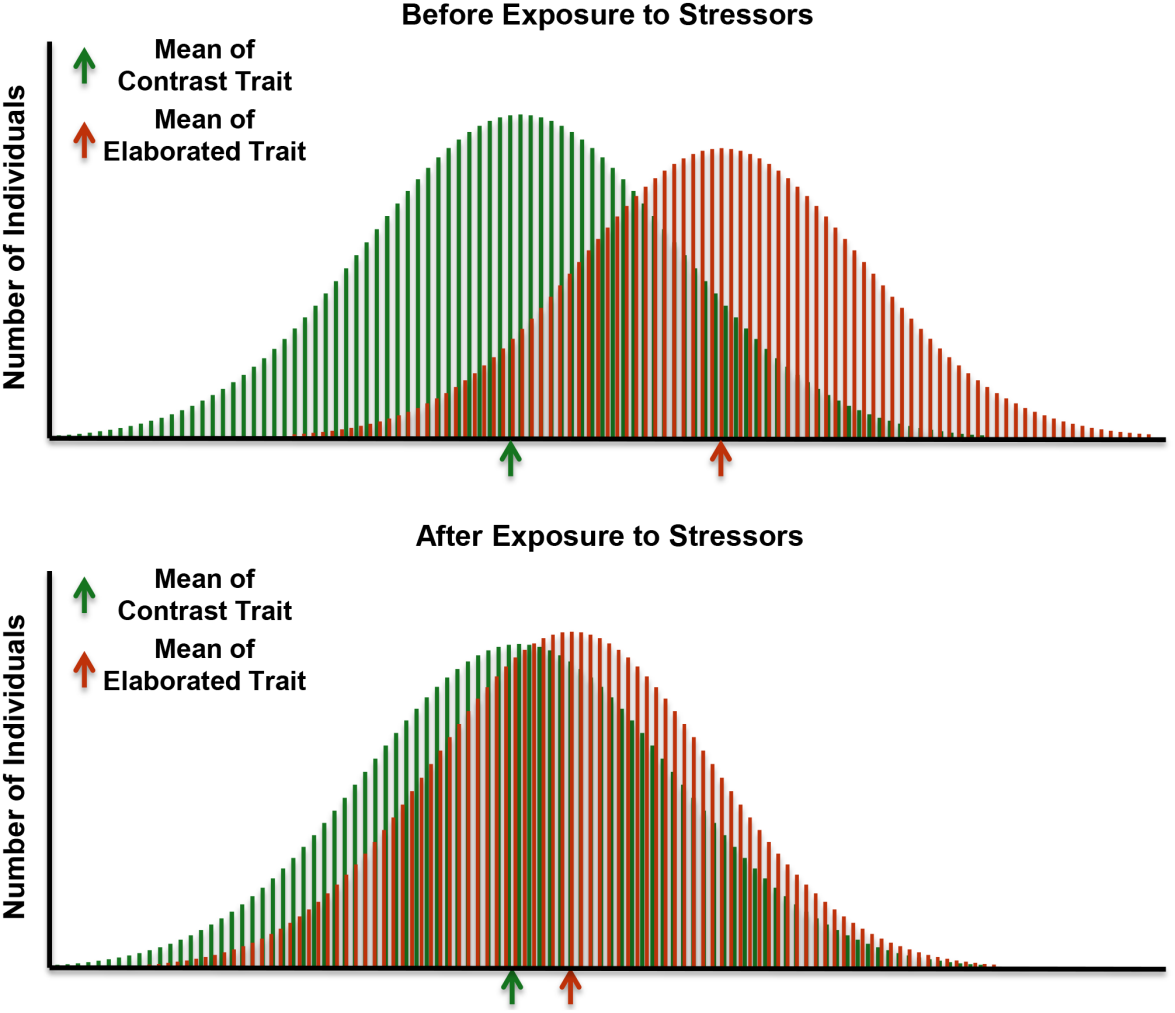
Sexual or social selection will result in the elaboration of traits that enhance or signal competitive abilities or influence mate choices. The top distributions show how these evolutionary processes result in larger traits (red) in one sex versus the other (green); or larger sexually selected (red) than naturally selected (green) traits in the same individual. As shown by the bottom distributions, exposure to parasites or to other stressors often has stronger effects on the elaborated than the contrast trait.

The peacock’s (*Pavo cristatus*) tail is one such trait, and is an indicator of immune system health [9]. The critical feature of these traits is that they are more sensitive to stressors than are other traits, such as the same trait in the opposite sex or a different, non-elaborated trait within the same individual. As shown in Fig 1, sexual or social selection (below) will result in the selective elaboration of one trait (red) over another (green) and a heightened sensitivity of the elaborated trait to stressors. We can reframe this concept—traits that have been elaborated through sexual or social selection have a heightened sensitivity to environmental and social stressors—to identify traits that are particularly vulnerable to disruption by NTDs and use them as barometers to gauge the efficacy of treatments for NTDs.

### Sexual and Social Selection

Darwin’s sexual selection [7] involves competition with members of the same sex over mates (intrasexual competition) and discriminative choice of mating partners (intersexual choice). These dynamics capture the evolution and expression of elaborated traits across hundreds of species [10], including our own [11]. The bulk of research on sexual selection has focused on male–male competition for mates and female mate choices, as these are very common dynamics and often result in the evolution of conspicuous physical, behavioral, or cognitive traits (below). There are many examples of female–female competition for mates and male choice [12–14], but these dynamics are less common, or at least less obvious, and thus not as well understood. As a result, sexual selection is more useful for identifying male-specific than female-specific vulnerabilities.

West-Eberhard [8], however, provided a perspective that should enable the identification of female-specific vulnerabilities; specifically, she proposed that competition for resources other than mates is a form of social selection and that sexual selection is a subset of these pressures. Both involve social competition for control of important resources; but in sexual selection, the resources at stake are mates, and in social selection, the resources at stake include access to food and nesting sites, among others. With this important insight, biologists have broadened the search for condition-dependent traits to include those that facilitate competition for access to reproduction-related resources, whether or not those resources are mates. This broader perspective provides considerable opportunity for female–female competition and the condition-dependent elaboration of traits that facilitate this competition [15,16].

#### Condition-dependent traits

Individuals with elaborated trait expression, as with the peacock’s tail, have competitive advantages over their peers or are often preferred as mates, but this does not tell us why these traits have a heighted sensitivity to stressors. The key idea is that these traits signal competitive abilities or qualities that will influence the wellbeing of their offspring [6,17–19]. In this circumstance, the benefits of cheating by expressing elaborated traits that are disconnected from their benefits (e.g., immune system genes), as well as the costs of being cheated, are high.

For instance, males in poor health (e.g., compromised immune system) may cheat by diverting resources (e.g., calories) to the development of these traits (e.g., elaborate tail plumage), thus bluffing other males from directly competing with them or enticing females to mate with them. Cheating can be reduced if the development and expression of these traits are costly to less fit individuals [20,21]. This is where sensitivity to stressors becomes important. Parasites are ubiquitous and can significantly compromise health and behavior. Some males, however, are better able to tolerate parasites than others, and those that tolerate parasites generally sire offspring that tolerate them as well [22,23]. Thus, it is in females’ best interest to choose mates that tolerate parasites and it is in these males’ best interest to signal parasite resistance [24]. In other words, for a reliable signal of parasite resistance to evolve, the expression of the trait must be modifiable by level of parasite infestation and must be elaborated to the extent that unfit males cannot fully express the trait and simultaneously cope with parasites [25]. This does not necessarily mean that fit males are free of parasites, but rather they are healthy enough to tolerate some level of infection and show only small to modest decrements in trait expression.

The result is the evolution of traits whose expression is dependent on environmental and social conditions. Some of these traits, such as bird song, are indirect signals; they are correlated with unseen traits, such as immunocompetence or the integrity of specific brain regions [26]. Other traits, such as the ability to navigate in the local ecology, are directly related to competition and are functional. Both direct and indirect signals are found in a spectacular variety of living organisms (e.g., [27,28]), and across this myriad of species, parasites are a key stressor that can compromise trait development and expression.

#### Parasites

Many elaborated traits are signals of the integrity of immune system defenses [24]. Indeed, parasites may have driven the initial evolution of sexual reproduction itself—by creating variability in the offspring’s immune system that then provides better defenses against local parasites [29]—which, in turn, explains why signals of parasite resistance are so common in sexually reproducing species. In a meta-analysis of studies of birds and mammals, Boonekamp, Ros, and Verhulst [30] found that immune challenges were consistently related to subsequent drops in male testosterone, and this, in turn, provides a mechanism linking parasite exposure to trait expression; specifically, drops in testosterone will compromise the expression of males’ sexually selected traits, such as the peacock’s tail [25]. The relation is actually more nuanced, as healthy males can tolerate high parasite loads and sport elaborated traits, but experimental increases in testosterone in their less healthy counterparts are associated with long-term increases in morbidity and mortality [31].

The complex relations between sex hormones, including estrogens and progesterone, and the varied immune functions leave abundant opportunity for similar processes to occur in females and in relation to other hormones [32,33]. Wedekind [34], in fact, suggested that different hormones or combinations of them may interact with immune functions in ways that result in parasite-specific suppression of exaggerated traits, with one trait signaling infestation with one type of parasite and other traits signaling other parasites. The extent of parasite-specific trait disruptions remains to be determined. In any case, the critical point is the discovery of female-specific traits that signal immune system functions [35–37].

### Parasite-Compromised Traits in Nonhuman Species

In this section, I provide a few illustrations of how parasitic diseases and other stressors can compromise the development and expression of specific traits. Generally, traits that involve anatomical elaboration, including skeletal structures and brain anatomy, are, in theory, most vulnerable when the trait is undergoing the most rapid changes [38], although the compromised nature of many of these traits may not be apparent until adulthood. Behavioral traits, such as courtship displays, tend to be energetically demanding and thus should be particularly susceptible to current stressors. Behavioral traits, as in fighting competence, may also be susceptible to developmental stressors to the extent that full expression is dependent on earlier developing brain systems or developmental practice of the behavior.

As noted, the most vulnerable traits can vary by sex, age, and even across closely related species, but are predictable with a sufficient understanding of the dynamics of competition and choice in the species. The basic idea and pattern can be illustrated by research on the relation between parasite exposure or immunocompetence and trait expression in birds, as shown in Table 1; the basic structure of the table and associated research provides the framework for studying human vulnerabilities (below). The studies are characterized as field experiments (F), laboratory experiments (L), or natural variation in a field setting (N). The field experiments involved some type of manipulation, such as experimentally inoculating some birds but not others and releasing all of them into their natural habitat to determine how exposure to common parasites influences trait development. The natural variation studies also involved assessment of animals in the field, but there was no experimental manipulation. The trait involved in competition or choice is then indicated and whether (yes, Y) or not (no, N) the study provided evidence that trait expression was compromised.

**Table 1:** Avian condition-dependent traits compromised by parasites.

Species	Scientific name	ST	Trait	CD	S	Contrast		Life History	Reference
						Trait	Effect		
**Color Traits**									
Red jungle fowl	*Gallus gallus*	L	comb	Y	M	tarsus length	C<T	postnatal/adult	[39]
Ring-necked pheasant	*Phasianus colchicus*	L	wattle	N	M	body size	C = T	postnatal	[40]
Goldfinch	*Spinus tristis*	L	beak	Y	B	female beak	C = T	adult	[41]
		L	beak	Y	M	body mass	C<T	adult	[42]
House finch	*Carpodacus mexicanus*	L	plumage	Y	M	NA	NA	postnatal	[43]
		L	plumage	Y	M	body size	C<T	postnatal	[44]
Diamond firetails	*Stagonopleura guttata*	L	plumage	Y	B	female plumage	C<T	adult	[45]
Greenfinch	*Carduelis chloris*	L	plumage	Y	M	inner feather	C<T	adult	[46]
Serin	*Serinus serinus*	N	plumage	Y	M	NA	NA	adult	[47]
		F	plumage	Y	M	NA	NA	adult	[47]
Great tit	*Parus major*	F	plumage	N	B	female color	C = T	adult	[48]
Blackbirds	*Turdus merula*	L	beak	Y	M	body mass	C<T	adult	[49]
Red jungle fowl	*Gallus gallus*	L	plumage	Y	M	tarsus length	C<T	postnatal/adult	[39]
Goldfinch	*Spinus tristis*	L	black cap	N	M	body mass	C>T	adult	[42]
House finch	*Carpodacus mexicanus*	L	tail	N	M	NA	NA	postnatal	[43]
Great tit	*Parus major*	F	black stripe	N	B	female color	C = T	adult	[48]
Wild turkeys	*Meleagris gallopavo*	L	plumage	Y	M	non-UV color	C<T	postnatal	[50]
**Size Traits**									
Red grouse	*Lagopus lagapus*	N	comb	Y	M	body size	C<T	adult	[51]
		N	comb	Y	F	body size	C = T	adult	[52]
		F	comb	Y	F	body size	C<T	adult	[52]
		F	comb	Y	M	NA	NA	adult	[53]
		N	comb	Y	M	NA	NA	adult	[54]
Red jungle fowl	*Gallus gallus*	L	comb	Y	M	tarsus length	C<T	postnatal/adult	[39]
Wild turkeys	*Meleagris gallopavo*	N	snood	Y	M	NA	NA	adult	[55]
Goldfinch	*Spinus tristis*	L	black cap	N	M	body mass	C<T	adult	[42]
Great tit	*Parus major*	F	black stripe	Y	B	female stripe	C = T	adult	[48]
Diamond firetails	*Stagonopleura guttata*	L	plumage spots	Y	B	male spots	C<T	adult	[45]
Collard flycatchers	*Ficedula albicollis*	F	forehead patch	Y	M	NA	NA	adult	[56]
Barn swallow	*Hirundo rustica*	N	tail length	Y	B	female tail	C<T	adult	[57]
**Behavioral Traits**									
Houbara bustard	*Chlamydotis undulata*	L	courtship display	Y	M	NA	NA	adult	[58]
Rock dove	*Columba livia*	L	courtship display	Y	M	body size	C<T	adult	[59]
Collard flycatchers	*Ficedula albicollis*	F	song rate	Y	M	NA	NA	adult	[56]
		F	song features	N	M	NA	NA	adult	[56]
Sparrow	*Zonotrichia leucophrys*	F	song rate	Y	M	body condition	C<T	adult	[60]
Great tit	*Parus major*	F	song duration	Y	M	body condition	C<T	postnatal	[61]
		F	song overlap	Y	M	body condition	C<T	postnatal	[61]
**Brain and Cognitive Traits**									
Atlantic canary	*Serinus canaria*	L	HVC volume	Y	M	brain volume	C<T	postnatal	[62]
		L	RA volume	N	M	brain volume	C = T	postnatal	[62]
		L	song repertoire	Y	M	NA	NA	postnatal	[62]
Sedge warbler	*Acrocephalus schoenobaenus*	N	song repertoire	Y	M	NA	NA	adult	[63]
Great tit	*Parus major*	F	song repertoire	N	M	NA	NA	postnatal	[61]

Note: ST = study type (L = lab experiment; F = field experiment; N = natural variation); CD = condition dependent (Y = yes; N = no); S = sex of animals included in the study (M = male; F = female; B = both sexes). Body condition refers to fat and muscle reserves. HVC is not an acronym but is sometimes termed higher vocal center. RA = robust nucleus of the arcopallium. Song repertoire is included under brain and cognition because it is a measure of song learning, whereas the song traits listed under behavioral have an energetic component to them.

As was illustrated in Fig 1, the condition-dependent trait should be more strongly compromised by parasites than other traits [4]. These contrast traits were measured along with the elaborated trait of interest and often included the same trait in the opposite sex or a similar trait within the same individual; studies that did not include contrasts are noted as NA (not applicable). If some type of potential contrast was available, it is noted in the table, along with whether or not parasites compromised the contrast trait less than the elaborated trait (C<T), more than the trait (C>T), or both were equally affected (C = T). Life history notes whether the exposure to parasites or the immune system manipulation occurred during development (postnatal) or in adulthood.

The red jungle fowl (*Gallus gallus*) illustrates how parasites can have selective effects on trait expression. Females of this species choose mates based in part on the red coloration and size of the males’ comb, and males with brighter and larger combs are typically socially dominant [19,64]. Zuk et al. [64] exposed jungle fowl chicks to a common intestinal worm (*Ascaridia galli*) and followed the development of comb size and color through adulthood. The size and color of the combs of parasitized males were compromised relative to that of unexposed males, but there were no differences in tarsus (i.e., “leg”) length. Moreover, females preferred unexposed to exposed males by 2:1. Zanollo et al. [45] found that different traits were indicators of immunocompetence in male and female diamond firetails (*Stagonopleura guttata*). Females have more white plumage spots than males, whereas males have a deeper red tail plumage color than females. Females but not males with more white plumage spots mount a stronger immune response than same-sex individuals with fewer spots. Males’ immune response is correlated with red plumage color, but females’ is not. Importantly, the sex-by-trait effect was only found during the breeding season, indicating a time-limited and hormone-dependent sensitivity of these traits.

Courtship displays, as noted, are energetically demanding and are very common social signals of health and vigor. These can range from repetitions of stereotyped behaviors to aspects of birdsong, and are consistently compromised by parasitic infections. Behavioral displays are generally good indicators of current condition [59], and song features can be indicators of current condition [60] or infection status during development [61]. For many species, bird song is acquired early in life and is dependent on the development of a specific system of brain regions [65]. Spencer et al. [62] found that juvenile canaries (*Serinus canaria*) infected with malaria (*Plasmodium relictum*) had not only poor song learning but also a compromised brain area critical to song learning and production. Song is not functional, but songbird males contribute to provisioning of offspring, and, thus, their ability to effectively forage should be one criterion that influences females’ mate choices. The brain system that supports foraging develops concurrently with the song system, and, thus, poor song quality should be correlated with poor foraging ability, which appears to be the case [66].

## Human Vulnerability

The examples above and many others indicate that there are not universal male-specific or female-specific trait vulnerabilities [4,67]. To be sure, there are many commonalities across species that result in similar traits being elaborated and, thus, potentially vulnerable in one or both sexes, but these can be understood in terms of similar selection pressures (e.g., territory expansion for mate searches) or common physiological mechanisms needed for trait elaboration or to cope with specific types of stressors [38]. The key to identifying sex-, age-, and trait-specific vulnerabilities is an understanding of the social dynamics that define competition and choice for the species.

Doing so for our species has a long and controversial history [68]. Alternative explanations for the many human sex differences that have been discovered over the past century [69] focus on nonphysical traits and social and social-cognitive processes [68,70]. Indeed, social factors such as the legal suppression of polygyny or parental biases (e.g., in provisioning) in the treatment of girls and boys can clearly have a substantial effect on the development and expression of some sex differences [11,71]; of course the most potent evolutionary process, natural selection, results in more similarities than differences between females and males [72]. Nevertheless, arguments that humans have somehow eluded the rigors of sexual and social selection are no longer tenable.

### NTDs and Physical Vulnerabilities

Physical male–male competition is consistently associated with larger males than females in terrestrial species [10]. On the basis of the fossil record, there has been a long history of larger males than females during hominid evolution. About 4 million years ago, our male predecessors of the species *Australopithecus anamensis* were at least 50% larger (likely more so) than our female predecessors [73], a pattern that continued with subsequent australopithecines [74]. The current differences emerged roughly 1.8 million years ago with *Homo erectus* [75]. The reduction in physical dimorphism may have been due to reduced polygyny and, thus, less intense competition, the emergence of male coalitions, or some combination [76]. Whatever the case, the current adult dimorphisms emerge from a pattern of development found in primates with intense male–male competition. In these species, males and females are more similar than different during the juvenile years, and males show an extended or exaggerated pubertal growth spurt when most of the adult differences emerge [77].

For humans, these dimorphisms include height as well as skeletal structure of the upper body, lean muscle mass, and cardiovascular fitness, among others [78]. Most of these differences are small to moderate during childhood and become quite large during adolescence. Correspondingly, sex differences in the vulnerability of these traits should be modest early in development and increase as boys and girls approach sexual maturity. There is, in fact, an expansive literature in physical anthropology and pediatrics for many of these sex differences, but previous studies and reviews did not take advantage of comparative insights and, as a result, often conflated stressors during childhood and adolescence [79]. The result would be an overestimation of sex differences in trait vulnerability during childhood and an underestimation during adolescence.

In addition to the timing of parasite exposure, it is important to consider other social and ecological stressors that could magnify or obscure the effects of parasitic infections. As briefly noted in the introduction and elaborated elsewhere [67], the two other natural stressors that consistently compromise the development or expression of elaborated traits are poor nutrition and social stress (e.g., as associated with low socioeconomic status [SES]). In other words, these traits have evolved to signal the individuals’ nutritional status and exposure to social stressors, not simply their exposure to parasites. We do not currently know whether different stressors (e.g., NTD versus poor nutrition) have differential effects on these traits, but it is likely that a combination of them will be particularly devastating, although still in a sex-, age- and trait-specific way. To fully understand these vulnerabilities, factors that moderate girls’ and boys’ and women’s and men’s exposure to these stressors need to be considered in combination with NTDs. For instance, any parental biases (whether favoring boys or girls) in the provision of nutrition or health care would need to be considered above and beyond my narrow focus on NTDs here. Any such biases will not only influence the risk of infection with NTDs but may be added stressors in and of themselves. The goal here is to highlight the sex- and age-specific nature of vulnerabilities to NTDs, not to downplay the importance of other potential stressors.

#### Height

One such vulnerable trait is boys’ height. As shown in Table 2, NTD exposure during childhood is sometimes found to compromise boys’ height more than that of girls [80] or uninfected boys [81], but there are just as many examples of no effect of NTD exposure. There is a more consistent pattern for studies that included children and adolescents together, but there are also several examples in which girls’ height appears to be more strongly compromised by NTDs than boys’ height. These reversals, however, may be related to other factors. For instance, Parraga et al. [82] found that the height of girls with schistosomiasis was compromised relative to uninfected girls, but infected and uninfected boys did not differ. Boys in both groups, however, were malnourished relative to girls, and their height was more severely compromised relative to same-sex norms than was the height of girls. As a result, the potential for further compromises in boys’ height was reduced. It was not clear why boys were poorly nourished relative to girls in this population, but the result is consistent with the vulnerability of boys’ height, albeit in this case not due directly to NTDs.

**Table 2:** Human condition-dependent physical traits disrupted by NTDs.

Parasites Assessed	ST	Trait	CD	S	Contrast		Life History	Reference
					Trait	Effect		
*Trichuris trichiura*	N	height	N	M	unexposed male	C = T	childhood	[88]
*Trichuris trichiura*; *Ascaris lumbricoides*; hookworm	F	height	Y	M	untreated male	C<T	childhood	[81]
*Ascaris lumbricoides*	F	height	N	B	female height	C = T	childhood	[89]
*Plasmodium falciparum*; *Schistosoma haematobium*; *Trichuris trichiura*; *Ascaris lumbricoides;* hookworms	N	height	Y	B	female height	C<T	childhood	[80]
*Schistosoma japonicum*	N	height	N	B	female height	C = T	childhood	[84]
*Schistosoma mansoni*; *Trichuris trichiura*; *Ascaris lumbricoides;* hookworm (*Ancylostoma duodenale*)	N	height	Y	B	female height	C<T	child/adolescence	[90]
*Schistosoma mansoni*; *Trichuris trichiura*; *Ascaris lumbricoides*	N	height	N	B	female height	C>T	childhood/adolescence	[82]
*Schistosoma mansoni*	F	height	Y	B	female height	C<T	childhood/adolescence	[91]
*Schistosoma haematobium*; *Trichuris trichiura*; *Ascaris lumbricoides; Fasciolopsis buski*	N	height	Y	B	female height	C>T	childhood/adolescence	[92]
*Schistosomiasis haematobium*; *Trichuris trichiura*; Hookworm; *Plasmodium falciparum*; *Wuchereria bancrofti*	N	height	Y	B	female height	C<T	childhood/adolescence	[93]
*Schistosoma haematobium*	N	height	Y	M	control male	C<T	adolescence	[83]
*Schistosoma japonicum*	N	height	Y	B	female height	C<T	adolescence	[84]
*Trichuris trichiura*; *Ascaris lumbricoides;* hookworm	F	muscle	Y	B	female muscle	C<T	childhood	[94]
*Ascaris lumbricoides; Schistosoma mansoni;*hookworm	N	muscle	Y	B	female muscle	C<T	childhood/adolescence	[95]
*Schistosoma mansoni*	F	muscle	Y	B	female muscle	C<T	childhood/adolescence	[91]
*Schistosoma japonicum*	N	muscle	Y	B	female height	C<T	adolescence	[84]
*Trichuris trichiura*; *Ascaris lumbricoides*; hookworm	F	fat reserve	Y	M	untreated male	C<T	childhood	[81]
*Schistosoma mansoni*	F	fat reserve	Y	B	female reserves	C<T	childhood/adolescence	[91]
*Trichuris trichiura*; *Ascaris lumbricoides;* hookworm	F	fitness	Y	M	untreated male	C<T	childhood	[96]
*Trichuris trichiura*; *Ascaris lumbricoides*	F	fitness	Y	M	untreated male	C<T	childhood	[81]
*Schistosoma haematobium*; hookworm	F	fitness	Y	M	untreated male	C<T	childhood	[97]
*Trichuris trichiura*; *Ascaris lumbricoides;* hookworm	F	activity	Y	B	female physical	C = T	childhood	[94]
*Schistosomiasis haematobium*	F	activity	Y^a^	B	female physical	C = T	childhood	[98]
*Trichuris trichiura*; *Ascaris lumbricoides*; hookworm	F	body condition	Y^b^	M	untreated male	C<T	childhood	[81]
*Schistosoma mansoni*; *Trichuris trichiura*; *Ascaris lumbricoides;* Hookworm; *Plasmodium malariae; P*. *falciparum*; *Entamoeba histolytica; Giardia intestinalis*	F	fitness	Y	B	female fitness	C<T	childhood/adolescence	[99]
*Schistosomiasis haematobium*; *Trichuris trichiura*; Hookworm; *Plasmodium falciparum*; *Wuchereria bancrofti*	N	fitness	N^c^	B	female fitness	C = T	childhood/adolescence	[93]
*Schistosomiasis haematobium*; *S*. *mansoni*; *Ascaris lumbricoides;* Hookworm; *Plasmodium*	N	fitness	N	B	female fitness	C = T	childhood/adolescence	[100]
*Trichuris trichiura*; *Ascaris lumbricoides;* hookworm	N	fitness	Y	B	female fitness	C<T	childhood/adolescence	[101]
*Schistosomiasis haematobium*;	N	fitness	N	B	female fitness	C = T	adolescence	[102]
*Entamoeba histolytica*; *Hymenolepis nana*; *Ascaris*; *Giardia*	N	sexual maturation	Y	M	NA	NA	adolescence	[103]
*Schistosoma mansoni*; *Trichuris trichiura*; *Ascaris lumbricoides*	N	fat reserve	Y	B	male reserves	C<T	childhood/adolescence	[82]
*Schistosoma mansoni*	F	fat reserve	Y	B	male reserves	C>T	childhood/adolescence	[91]
*Schistosoma haematobium*; *Trichuris trichiura*; *Ascaris lumbricoides; Fasciolopsis buski*	N	fat reserve	Y	B	male reserves	C = T	childhood/adolescence	[92]
*Schistosoma japonicum*	N	fat reserve	Y	B	male reserves	C<T	adolescence	[84]
*Onchocerca volvulus*	N	skin health	Y	F	NA	NA	adult	[104]

Note: ST = study type (F = field experiment; N = natural variation); CD = condition dependent (Y = yes; N = no); S = sex of participants or for trait of interest (M = male, F = Female, B = both sexes).

^a^Treatment with antiparasite increased the physical activity of both sexes. The effect was larger in boys than girls, but it was not statistically significant.

^b^Body condition is weight controlling for height (an estimate of fat and muscle reserves), a common measure of physical condition in biological field studies.

^c^Parasite loads were not directly related to fitness as measured by a run test, but they were related to poor nutrition and stunting, which were related to physical fitness.

The critical studies are those that examine compromised height in adolescence, and both of these indicated that schistosomiasis compromises boys’ height relative to that of uninfected boys [83] or infected girls [84]. Related studies confirm that during adolescence, boys’ height is more strongly compromised than that of girls when growing up in lower status households [85], and as a result of poor nutrition [86] and anemia [87].

#### Fitness and muscle mass

**Fig 2:**
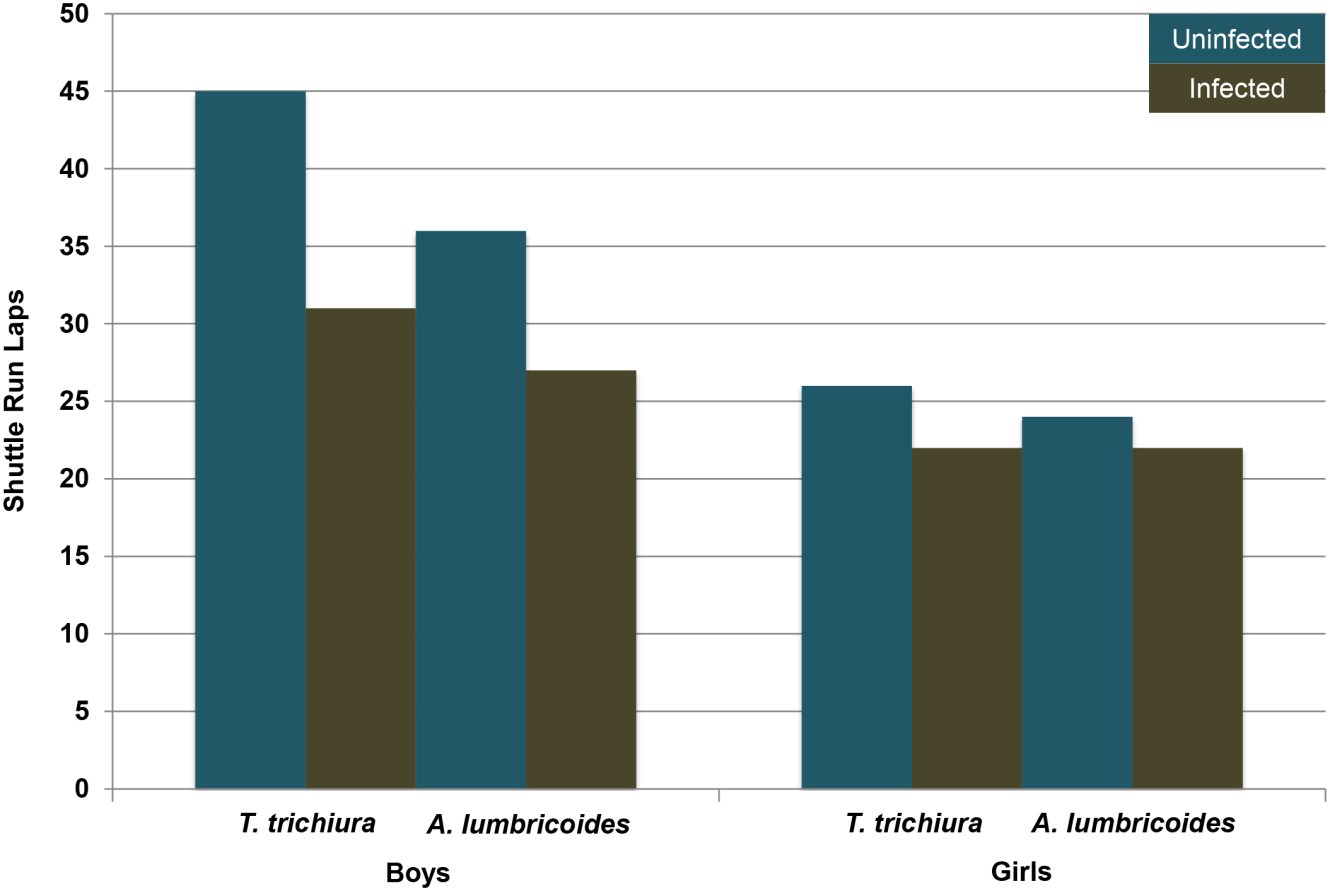
Infection with two common NTD parasites compromises boys’ fitness more strongly than that of girls. The results are based on the commonly used shuttle run measure, whereby fitness is determined by the number of 20-meter back-and-forth laps that can be completed in a fixed amount of time. The figure is based on data (combined across children and adolescents) presented in [101].

In contrast to height, boys’ physical fitness, activity levels, and lean muscle mass may be more vulnerable to NTDs and other stressors from childhood forward; these vulnerabilities will be greater in adolescence and adulthood than in childhood. During childhood, the sex differences for these traits are larger than those found for height [105,106], and, more critically, are likely related to sex differences in one-on-one and coalitional play; that is, developmental preparation for male–male competition in adulthood (below). The majority of studies reviewed in Table 2 are consistent with this prediction. As illustrated in Fig 2, Yap et al. [101] found that exposure to two common parasites (*Trichuris trichiura*; *Ascaris lumbricoides*) compromised the physical fitness of boys (children and adolescents combined) more strongly than that of girls, with particularly large effects for *T*. *trichiura* (see also [107]). Stephenson and colleagues [81,96], in contrast, found evidence for relatively larger effects of *A*. *lumbricoides* than *T*. *trichiura* on boys’ fitness. The mixed results may be due to the multiple influences of these parasites and comorbid difficulties. For instance, Bustinduy and colleagues [93] did not find a strong relation between parasite infestation and children’s and adolescents’ fitness, but parasite levels were related to stunting and anemia—and more strongly in boys than girls—which, in turn, did compromise fitness. In this case, the effect of NTDs on boys’ fitness was mediated by small stature and anemia. Whatever the mechanisms, the main point is that the overall trend is for NTDs to compromise boys’ fitness and activity levels more severely than those of girls.

#### Fat reserves

Another common measure of health and growth is fat reserves, but the relation between this trait and NTDs (and other stressors) may be subtle. Toward the end of childhood, boys, but not girls, show an increase in upper arm body fat that may provide an important reserve to support the early phases of pubertal growth and, as a result, may be a sensitive indicator of boys’ condition in late childhood [108]. Girls, in contrast, show steady increases in arm fat and exceed that of boys during adolescence and adulthood [108]; overall, women need a large fat reserve to become pregnant and to support breastfeeding [109]. The one study that focused on NTDs in children found that boys’ fat reserves were indeed more compromised that those of girls [81], whereas the one study of adolescents found that girls’ fat reserves were more compromised than those of boys [91]. The remaining studies combined children and adolescents, with mixed results.

The mixed results likely stem from variation in the onset of girls’ pubertal growth spurt that, in turn, will influence age-related estimates of the relative vulnerability of the fat reserves of boys and girls. Kulin et al.’s [110] study of the relation between SES and fat reserves illustrates the issue. At 10 years of age, Kenyan boys from higher SES families had more triceps fat reserves than girls from the same background, but thereafter, boys’ reserves dropped and girls’ increased. Girls from lower SES families did not show gains in fat reserves until three years after their better-off same-sex peers, and even then the increases were relatively modest; the fat reserves of boys from lower SES backgrounds were consistently low across age. The overall result was a larger fat reserve deficit for 10-year-old boys than girls, a sex difference that was reversed for 13-year-olds. Critically, if the study had only included low SES children, the fat reserve deficit of girls would not occur until later in adolescence.

The issue is further complicated by age-differences in trade-offs between use of fat versus muscle reserves to cope with nutritional shortfalls. Reiches et al. [111] assessed these trade-offs for adolescent girls and young women in Gambia across seasons of high versus low food availability. Younger, still-growing girls lost more fat than muscle during the “hungry” season, whereas older girls and young women who were no longer growing lost smaller amounts of fat but larger amounts of muscle, a pattern that would better support pregnancy and breastfeeding. Despite these caveats, triceps fat reserves may provide a useful indicator of boys’ condition in late childhood and girls’ condition thereafter. Critically, studies of age and sex differences in the sensitivity of fat reserves to stressors and any other physical or cognitive traits that change rapidly during adolescence will be more reliable if the trait under study is calibrated to stage of pubertal development and not simply age; the latter can be easily assessed for both sexes using Marshall and Tanner’s [112,113] scales.

#### Pelvic development

Finally, from an evolutionary perspective, pelvic development might be a trait of heightened vulnerability in adolescent girls, with an attendant increase in later risk of birth complications [114,115]. Over evolutionary time, women’s pelvic width expanded to accommodate increases in neonates’ cranial volume and, thus, may be more strongly related to natural than sexual selection [114]. Still, the associated waist-to-hip ratio does influence male choice [116], and, thus, sexual selection likely had some influence on the enhancement of pelvic width. I could not find studies of NTD exposure and pelvic development. Studies of other stressors (e.g., malnutrition) that included both height and pelvic width suggest that stressors do not compromise girls’ pelvic development more than their height or boys’ pelvic development during childhood [117].

**Fig 3:**
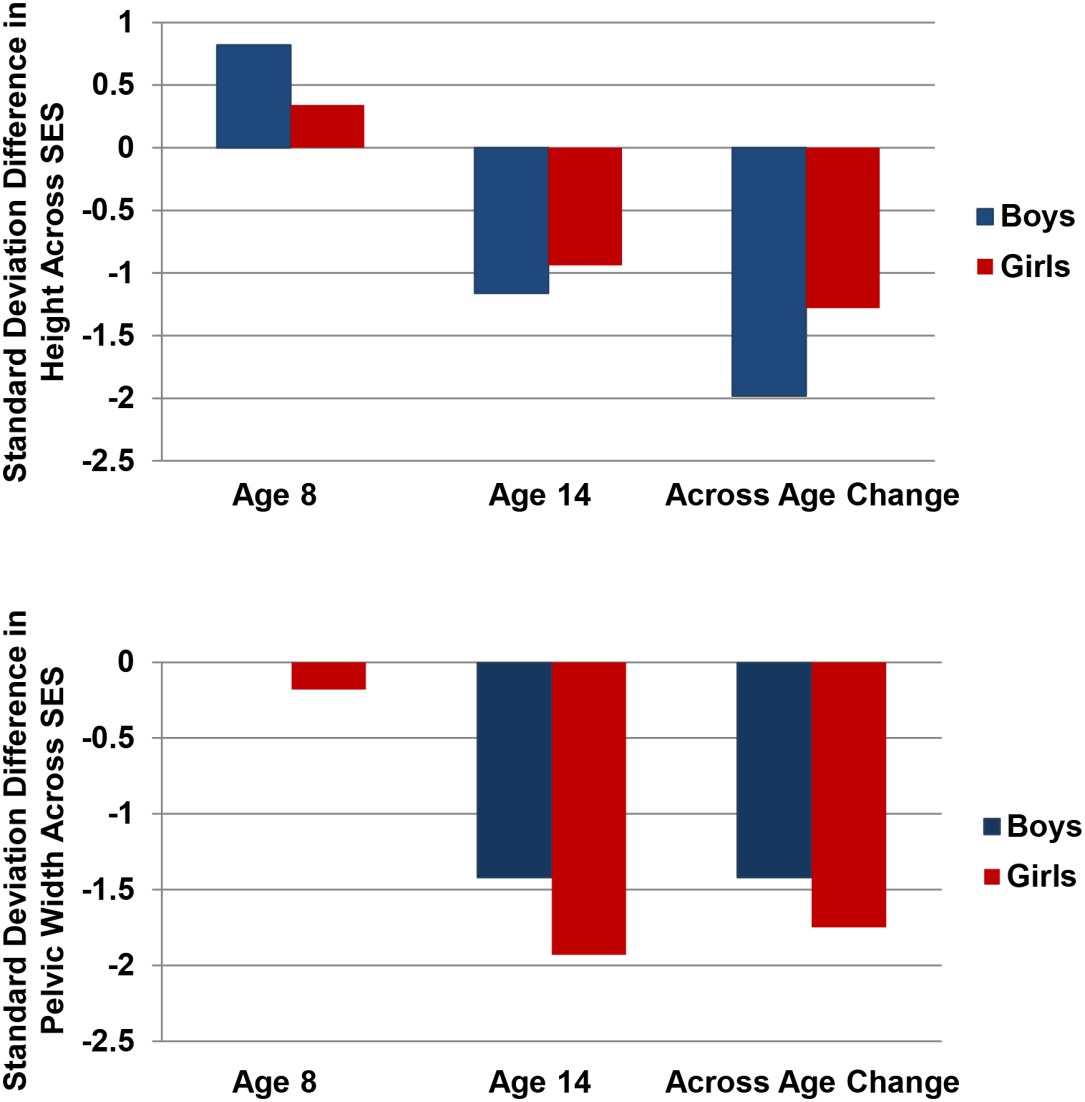
In developing populations, the stressors associated with low socioeconomic status (SES) compromise boys’ height more than that of girls and girls’ pelvic development more than that of boys. The figure shows standard deviation (SD) differences (lower SES—higher SES) in height and pelvic width for 8- and 14-year-olds from lower SES and higher SES families from Tanzania. Values > 0 indicate children from lower SES families scored higher, whereas values < 0 indicate children from lower SES families scored lower. Eight-year-olds from lower SES families were somewhat taller than their higher SES peers, but this reversed for 14-year-olds. Critically, the SES differences across age were larger for boys’ height (SD = 1.98) than pelvic width (SD = 1.42), and were larger for girls’ pelvic width (SD = 1.75) than height (SD = 1.28). The figure is based on data presented in [118].

There are too few studies of adolescents to draw firm conclusions, but the story may be different here. In one such study, Hautvast [118] assessed the physical development of Tanzanian children and adolescents from wealthier and poorer communities. He did not explicitly assess NTDs, but lower-status individuals are more likely to be infected than are their higher-status peers [2]. To illustrate potential sex-, age-, and trait-specific effects on physical growth, I calculated (in standard deviation units) differences in height and pelvic width for 8- and 14-year-olds from lower and higher SES families. The top section of Fig 3 shows that 8-year-old boys and girls from lower SES families were taller than their higher SES peers (bars in Fig 3 > 0), but this pattern was reversed for 14-year-olds (bars in Fig 3 < 0). The relative change across the 8- and 14-year-olds shows that the height of boys is more severely compromised by growing up in a low SES family than the height of girls, consistent with earlier discussion. The bottom section of Fig 3 shows little difference in the pelvic widths of 8-year-olds from lower- and higher SES families but, again, a substantive deficit for 14-year-olds from lower SES families. Critically, and in contrast to height, the pelvic width of girls is more severely compromised than the pelvic width of boys. In a similar study of adults from India, men and women growing up in lower SES families were found to be shorter and with narrower pelvic widths than their better-off peers. But, men’s height was more affected than their pelvic width, and women’s pelvic width was more affected than their height [119]. However, the latter differences were small and not tested statistically. Although in need of replication, the results suggest that adolescent girls’ pelvic development may be particularly sensitive to stressors, including, perhaps, NTDs.

### NTDs and Potential Behavioral and Cognitive Vulnerabilities

There are many behavioral, social, and cognitive traits that were very likely related to competition and choice during human evolution and, thus, may show heightened sex- and age-specific vulnerabilities [11,67]. It is not feasible or likely necessary to measure all of them, but assessing a few key traits may enable a broader study of the potential consequences of NTDs. These key traits would include play and social relationships in children and adolescents and social and visuospatial cognition in children, adolescents, and adults.

#### Play and social behavior

For mammals, play may enable the adaptation of specific physical, behavioral, or social competencies to the nuances of the local ecology, and, for some forms of play (e.g., locomotor), there are no consistent sex differences across species [120,121]. Exceptions to this pattern are play fighting and parenting. Sex differences in play fighting track differences in the intensity of physical competition in adulthood [121], and, for primates, sex differences in play parenting track differences in later investment in offspring [122]. Sex differences in children’s play follow a similar pattern, with boys engaging in more rough-and-tumble play and girls in more family-oriented play. These differences begin to emerge around two years of age and are influenced by prenatal and early postnatal exposure to sex hormones [123,124]. The sex difference in overall engagement in boy-typical (including play fighting) and girl-typical (including play parenting) play is quite large (*d* ~ 2) [125], and can be easily measured through parental report for preschool and older children (see [125,126]). The items may need some adjustments to local populations and opportunities to play, but the sex differences are so robust that any adjustments should not undermine the assessments. Items focusing on boys’ engagement in rough-and-tumble and group-level competitive play and girls’ play with child substitutes (e.g., dolls or fruit used like a doll) may be particularly sensitive.

Children’s **e**ngagement in sex-typical play may be important for their early social development, enabling them to integrate into same-sex social groups and to form same-sex social relationships. Boys’ tend to form larger, better integrated social networks than girls, and cooperate in the context of these groups, especially when competing against other groups [127]. Girls, in contrast, form closer dyadic relationships that serve as buffers against social stressors [128]. The latter can be assessed using a questionnaire [129]. Integration within a social network (all members know and like one another) requires a sociometric analysis [130], but can probably be inferred by the amount of time boys spend playing or competing within a stable social group [131].

These forms of play and relationships can have long-term social and psychological consequences, especially for boys. Relative to children who engage in sex-typical play, boys who engage in sex-atypical behaviors (e.g., doll play) are treated more negatively by same-sex peers, and often by adults, than are girls who engage in sex-atypical behaviors (e.g., rough-and-tumble play) [132,133]. Young and Sweeting [134] found that adolescent boys who did not engage in sex-typical behaviors were more victimized, socially isolated, and had more depressive symptoms than did girls who did not engage in sex-typical behaviors; in fact, the sex-atypical and -typical girls did not differ socially or psychologically from one another (see also [135]).

In other words, stressors that disrupt children’s sex-typical play and social behaviors can have long-term consequences. Although there is much that remains to be learned, there is evidence that some types of stressors, including toxin exposure and malnutrition, can have sex-specific effects on these play and social behaviors [136,137]. Given their sex-specific sensitivities and that they presage later social and psychological functioning, including measures of these traits in the study of NTDs may be informative. Equally important, sex differences in these types of behaviors are evident and easily assessed in children in developing nations (see [136]).

#### Cognition

To identify cognitive traits that are more likely to show sex- and age-specific vulnerabilities, it is important to distinguish between evolved and universal abilities (e.g., language, spatial navigation) and those that are culture-specific and largely dependent on formal schooling (e.g., academic mathematics) [138]. The same caveat applies to social behaviors; that is, the utility of this approach is strongest for universal behaviors (see [11]). Still, deficits in evolved abilities can compromise learning in school and in occupational settings to the extent this culturally-specific learning is dependent on evolved abilities; for instance, the ability to learn how to read and write is dependent on the evolved language system. With respect to evolved abilities, girls and women generally have advantages for folk psychological areas—language, sensitivity to nonverbal social cues, theory of mind (making inferences about the thoughts and feelings of others)—that support dyadic relationships and discourse, and boys and men generally have advantages for folk physics areas—navigating, tracking objects moving through space, detecting embedded objects—that support engagement with the physical world [139–142].

Geary et al. [143] proposed that girls’ and women’s advantages in these folk psychological domains are related to relational aggression, that is, subtle manipulation of relationships (e.g., by spreading false rumors) in the competition for mates or other resources [144], and the development and maintenance of friendships that serve as buffers against these and other stressors [145]. Indeed, relational aggression increases, especially in girls, during adolescence [146] and is likely over real or perceived romantic partners. Ethnographic accounts also suggest women use relational aggression to gain access to their husbands’ resources in the context of polygynous marriages that are common in non-Western cultures [147]. Boys’ and men’s advantages in spatial abilities are consistent with range sizes that are two to four times larger than those of girls and women in traditional societies, as associated with hunting and between-group contacts, including warfare [148,149]. The earlier noted sex differences in the skeletal structure of the upper body are consistent with men’s use of projectile (and blunt force) weapons, and their cognitive advantages are associated with enhanced skill in the targeting of projectiles thrown at objects [141] and the defensive evasion of projectiles hurled at them [150]. The latter is important because it is more consistent with male–male competition than hunting; hunting likely emerged later in our evolutionary history (see [11]).

Unfortunately, there is little research on the relation between NTDs and sex differences in these cognitive competencies, but there is evidence for sex-specific disruptions to these traits with exposure to other natural stressors (i.e., poor nutrition, social stress) and to manmade toxins. Across species, traits that show a heightened sensitivity to these stressors also tend to show a heightened sensitivity to parasitic infection [67].

To illustrate, there is evidence that girls’ and women’s language competencies are more sensitive to disruption from a variety of stressors than the same competencies of boys and men [151,152]. Overall vocabulary is not a useful measure of this sensitivity, but measures of the complexity of language comprehension and production (e.g., the grammatical structure of utterances) that are available in standardized tests do appear to be useful [153]. Stressors can also compromise the ease with which girls and women retrieve words from long-term memory. These potential deficits can be assessed with standardized measures [154], but should also be assessable with informal measures. The latter could include the number of category-related words that can be generated in one minute, such as the number of names of fruits or animals that can be retrieved from long-term memory [155]. The speed and accuracy of naming common objects might also be useful; these should be common to the local community but vary in how often they are seen and used, to create variation in level of difficulty.

There is also some evidence that other aspects of girls’ and women’s social-cognitive competencies can be compromised by exposure to stressors [156]. These competences include accuracy in inferring the emotions signaled by facial expressions, body posture, and vocal intonation. There are standard experimental tests of these competencies, but these require a computer and specialized software to administer [157]. There are also low-technology measures that should be easy to administer in field settings [158,159]. Girls and women also have advantages in memory for faces and perhaps especially the faces of other girls and women [160,161]. There are experimental measures of this form of memory, but it might also be assessed in the field using a set of facial photographs. Specifically, presenting a series of photos of faces of men and women (or boys and girls) one at a time to the individual, and then, after a short break (5 to 10 minutes), presenting half of these again intermixed with an equal number of new photographs. Memory will be reflected in the number of faces recognized as being in the first group minus the number of new faces falsely “recognized.”

**Fig 4:**
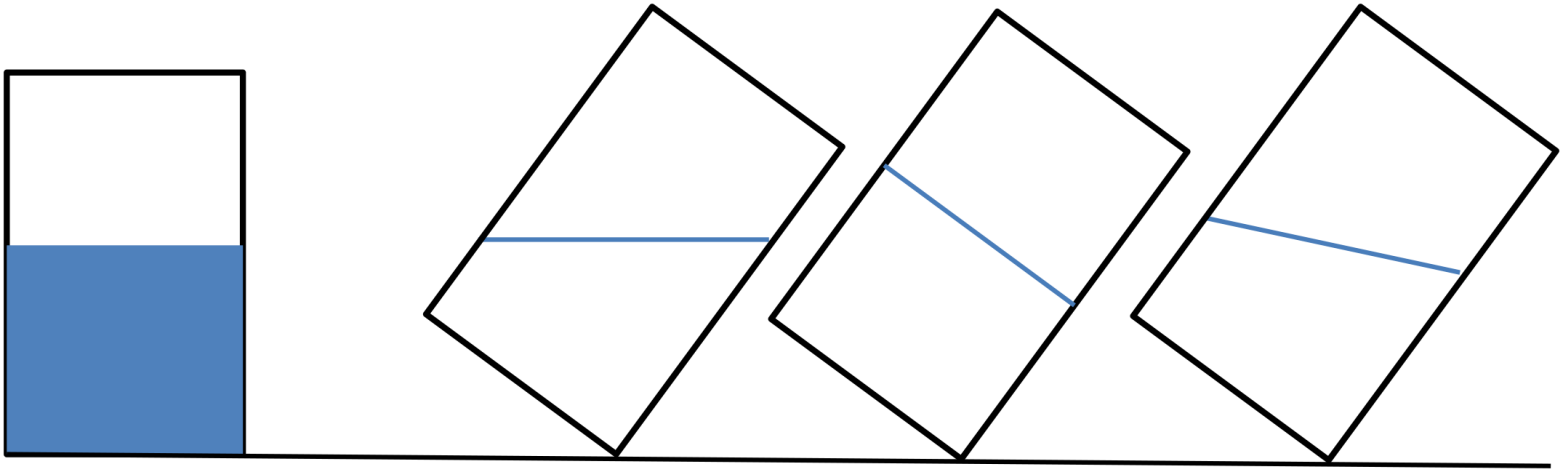
The water level task is a common measure of spatial reasoning. The goal is to determine the level of the water, after the glass is tipped. The task can involve asking people to draw a line showing this level in an empty glass, with the score as the degree of deviation from horizontal. Options can also be presented for people to choose among, as shown here.

There are many measures that assess the types of folk physics abilities that are likely to be more vulnerable to stressors in boys and men than girls and women, and several of these have been used successfully in field studies of the Hadza (Tanzania) and Twe and Tjimba (Namibia, Angola) [148,162]. One type of task generally assesses the ability to mentally represent and transform images of objects, such as recognizing what a three-dimensional shape will look like when rotated 90 degrees or determining water level in a tipped glass, as shown in Fig 4 (see [163,164]). Other tasks assess navigational ability, which can be assessed in the field by asking people to point to the location of distant villages or other known but not regularly traveled to locations (see [148]). Still other easy-to-use tasks would involve accuracy in targeting at varying distances; an underhanded beanbag toss can be used here. Similar measures are available for use with children [165]. Importantly, performance on these types of measures is correlated with men’s range sizes and reproductive success in traditional societies [148,162].

Critically, performance on these types of tasks and more complex spatial reasoning measures appears to be more easily disrupted by exposure to a variety of stressors in boys and men than girls and women. The sex-specific sensitivity of spatial abilities has been found with prenatal exposure to toxins [166] and toxin exposure during childhood and in adulthood [167,168]. Boys’ but not girls’ spatial abilities have also been shown to be compromised by growing up in lower SES families [169]. The sensitivity of these abilities to NTDs has not been directly assessed, as noted, but there is suggestive evidence.

Venkataramani [170] examined the spatial reasoning of adults from regions of Mexico with high rates of infection with malaria (*Plasmodium vivax*) before and after eradication of disease-carrying mosquitos. Men born after the eradication had higher spatial reasoning abilities than those born before the eradication, and the spatial reasoning gap between men from high- and low-infestation regions declined 25% to 51% post-eradication, controlling other factors (e.g., regional wealth). Eradication had no effect on women’s spatial reasoning or men’s success in school, suggesting sex- and trait-specific effects. At the same time, it must be noted that these results cannot be attributed to reduction in malarial infections with certainty, because mosquito eradiation was coincident with improvements in infrastructure and general health care that could also improve cognitive outcomes. Whatever the contributing factors, the critical finding is men’s spatial reasoning improved and that this improvement did not, at least in this study, generalize to school outcomes, suggesting selective benefits within men, along with the sex difference in improvement in spatial cognition.

## Discussion

The coevolution of parasites and hosts’ defenses against them may have driven the initial evolution of sexual reproduction [29]. Once sexual reproduction evolved, the stage was set for the evolutionary elaboration of traits that facilitate social competition for mates or access to other key reproductive resources (e.g., nesting sites) and discriminative choice of mating partners [7,8]. It is not too surprising, then, that the coevolving relation between parasites and hosts’ defenses is now interlocked with the proximate development and expression of many of these elaborated traits [25]. For some species, there is evidence that some parasites (e.g., *Toxoplasma gondii*) may actually enhance these traits and do so in ways that may benefit the parasite (e.g., promoting transmission) [171,172], but generally, parasitic infections compromise trait development and expression; nevertheless, the study of host-parasite interactions from the perspective of the parasites’ best interest and as related to sexual and social selection is an avenue for future study. Overall, the result is hard-to-fake signals of the individuals’ exposure and resilience to parasites, among other stressors [6]. These signals then guide the dynamics of competition and choice in natural populations, but can also be reframed and used to add nuance to the study of human vulnerabilities to parasites, including NTDs and other stressors [67].

The value added by this perspective is the potential to better identify the traits that are most likely to be compromised by NTDs and when in development, and for which sex, these traits are most likely to be disrupted. Without this knowledge, the search for specific vulnerabilities is much like searching for a needle in a haystack. We may assess traits that are not strongly affected by parasites, miss those that are affected, or assess the right traits but at the wrong time or in the wrong sex. The result is the potential to underestimate the consequences of stressor exposure or even determine that exposure has no harmful consequences at all when there is in fact a risk. The task of identifying vulnerabilities is further complicated because exposure to stressors early in development may not manifest until adulthood. The framework I have outlined here can be used to design broader assessments of the consequences of exposure to NTDs and, thus, capture some of these nuances.

To illustrate my point, consider Assis and colleagues’ [91] field study of the effects of treatment for schistosomiasis on the physical development of 7- to 14-year-old boys and girls. The children were randomly assigned to treatment and placebo control groups, and their physical growth was followed for the next year. One year after treatment, treated boys were taller, weighed more, and had larger fat reserves and more muscle mass than their untreated peers, but there were no differences in the growth of treated and untreated girls. On the basis of these results, we might conclude that this NTD is not particularly harmful for girls. This, however, would be a premature conclusion, if schistosomiasis compromises different physical traits in girls than boys. As described earlier, these traits might include pelvic width and fat reserves for adolescent girls; Assis et al. combined across ages and, thus, the latter could not be assessed in this study. Furthermore, the study leaves unanswered the questions of whether and how schistosomiasis differentially affects the sex-typical play, behavioral and social development, and specific cognitive competencies (e.g., reading facial expressions versus navigating a maze) of girls and boys. Given the sex- and age-specific vulnerabilities of many of these traits to other types of stressors (e.g., poor nutrition, social stress), the potential for NTDs to compromise them in nuanced but predictable ways might be considered.

These traits might also be useful for evaluating the efficacy of chemotherapy for NTDs. Because elaborated traits will be the first to be compromised, they are also the most likely to benefit from treatment. The rapidity and extent to which these traits respond to treatment might then be a useful indicator of the relative efficacy of one treatment or another and provide a priori predictions about how treatment will boost wellbeing. For many physical traits that involve skeletal growth, for instance, treatment is likely to be most beneficial during ages of most rapid growth—early infancy and adolescence—for both sexes but for different traits. One implication is that the relative costs of foregoing treatment in childhood may be small relative to foregoing treatment in adolescence, but this may not apply to other traits in the same way it does to skeletal growth. This is because periods of slow physical growth are also periods of rapid brain development in humans [173]. The full consequences of treatment decisions and associated benefits can only be evaluated based on a broad view of any cost-benefit tradeoffs, and this is where the inclusion of behavioral, social, and cognitive traits in the study of NTDs might be practically useful.

The approach I am advocating here might also be useful for identifying vulnerabilities that are similar for boys and girls, based on Hill’s [38] proposal that the efficiency of cellular respiration and control of oxidative stress are the fundamental biological mechanisms underlying the heightened vulnerability of sexually selected traits. If these are fundamental mechanisms, then they should apply to all traits—not just those associated with competition and mate choice (i.e., sexual and social selection)and to both sexes during the developmental period when the trait is undergoing rapid change. My focus here and in the literature on sexual selection follows from this broader perspective, because these traits generally undergo more rapid or prolonged development than other traits (e.g., peacocks’ tail versus peahens’ tail) or are energetically demanding (e.g., courtship displays). However, if we extend this approach to traits not directly related to competition and choice, we may be able to identify vulnerable periods and traits that are common to both sexes. I noted brain growth during childhood occurs more rapidly during periods of slow physical growth and vice versa [173]. Rapid brain growth is necessarily metabolically demanding, and any stressor, such as NTDs or poor nutrition, that compromises cellular respiration should, in theory, also compromise overall brain development in girls and boys, above and beyond brain systems (e.g., language versus spatial cognition) associated with competition and choice.

Among the many unanswered questions are whether different NTDs compromise different traits; whether the effect of NTDs on trait development and expression is comparable to the effects of other stressors (e.g., malnutrition) or differs from these; and how NTDs interact with other stressors. Despite these unanswered questions, the basic phenomenon of condition-dependent trait expression is well established for nonhuman species (e.g., [4]). The same basic principles are applicable to humans and provide a nuanced approach to the study and prevention of sex-, age-, and trait-specific vulnerabilities that is not available with alternative conceptual frameworks. The full realization of the benefits of the approach I am advocating here will require multidisciplinary teams that draw on expertise from psychologists, psychiatrists, neuroscientists, parasitologists, biologists, epidemiologists, and clinicians.

**Key Learning Points**

- **Traits that support competition for mates or other key resources or influence mate choices are generally elaborated relative to other traits.**
- **Elaborated traits have evolved such that they signal the individuals’ exposure and resilience to environmental and social stressors, including parasites.**
- **The sensitivity of these traits to disruption by parasitic infection is found across sexually reproducing species.**
- **Applying the concept to humans should enable the identification of sex- and age-specific vulnerabilities in traits that can be easily compromised by exposure to neglected tropical diseases.**

**Five Key Papers in the Field**

1. **Cotton S, Fowler K, Pomiankowski A. Do sexual ornaments demonstrate heightened condition-dependent expression as predicted by the handicap hypothesis? Proc Biol Sci. 2004;271: 771–783.**
2. **Zahavi A. Mate selection-a selection for a handicap. J Theor Biol. 1975;53: 205–214.**
3. **Hamilton WD, Zuk M. Heritable true fitness and bright birds: a role for parasites? Science. 1982;218: 384–387.**
4. **Folstad I, Karter A. Parasites, bright males, and the immunocompetence handicap. Am Nat. 1992;139: 603–622**
5. **Hill GE. Cellular respiration: the nexus of stress, condition, and ornamentation. Integr Comp Biol. 2014;54: 645–657.**
